# Predictors of Mortality in Peripheral Arterial Disease After Endovascular Lower Limb Revascularization and Development of a Risk Score Based Solely on Clinical Presentation

**DOI:** 10.3390/jcm15041364

**Published:** 2026-02-09

**Authors:** Gladiol Zenunaj, Lorenzo Ciofani, Luca Erbazzi, Aaron Thomas Fargion

**Affiliations:** 1 Unit of Vascular and Endovascular Surgery, Thoraco-Cardio-Vascular Department, University Hospital of Ferrara, 44121 Ferrara, Italy; aaron.fargion@ospfe.it; 2 Department of Translational Medicine for Romagna, University of Ferrara, 44121 Ferrara, Italy; lorenzociofani95@gmail.com (L.C.); l.erbazzi@hotmail.it (L.E.)

**Keywords:** peripheral arterial disease, endovascular revascularization, mortality, prognostic model, clinical risk score, chronic limb-threatening ischemia, tissue loss

## Abstract

**Background**: Patients with peripheral arterial disease (PAD) undergoing endovascular revascularization remain at high risk of long-term mortality. While anatomical characteristics such as the length of the lesion, chronic total occlusions and multi-segmental distribution strongly influence revascularization strategy and limb outcomes, their prognostic impact on survival is less clearly defined. The combination of clinical comorbidities, clinical limb presentation and anatomical factors may help to better predict mortality rate before endovascular lower limb revascularization. The primary endpoint of this study was to identify independent predictors of mortality in PAD patients, and the secondary endpoint was to develop a simple clinical risk score for individualized prognostic stratification. **Methods**: We conducted a single-center retrospective observational study including 476 consecutive PAD patients undergoing endovascular revascularization over a 6-year period. The endpoint target considered was all-cause mortality. Cox proportional hazards regression was used to identify independent predictors of mortality. A prognostic model was derived and subsequently simplified into a point-based clinical risk score (GZ-PAD Mortality Score). Model performance was assessed within the study cohort using Kaplan–Meier survival stratification and receiver operating characteristic (ROC) analysis. **Results**: Multivariable analysis identified age (HR 1.041 per year, *p* < 0.001), coronary artery disease (CAD) (HR 1.56, *p* < 0.001), chronic kidney disease (CKD) (HR 1.52, *p* = 0.038), dialysis dependence (HR 2.50, *p* < 0.001), and tissue loss (Rutherford 5–6; HR 5.33, *p* < 0.001) as independent predictors of mortality, whereas anatomical variables such as lesion length, chronic total occlusions and poor run-off vessel lost prognostic significance. For each patient, the linear predictor (XBETA) was calculated from the coefficients of the final Cox regression model and used to build the mortality score. Based on the 33rd and 66th percentiles of the XBETA distribution, patients were stratified into three prognostic categories: low risk (XBETA ≤ −0.43081), moderate risk (−0.43080 to 0.50835), and high risk (>0.50836). Kaplan–Meier analysis showed a significant discrimination (log-rank χ^2^ = 102.441; *p* < 0.001) and good discriminative performance (AUC 0.752). Next the model based on the XBETA predictor was simplified into the global ischemic-systemic risk score (GZ-PAD) assigned for ages 60–69 years = 1 point; 70–79 years = 2 points; ≥80 years = 3 points, CAD = 2 points; CKD = 1 point; Dialysis dependence = 3 points; and tissue loss = 6 points. The new model assessed with survival curves provided robust risk stratification across low-, moderate-, and high-risk groups (log-rank χ^2^ = 88.883; *p* < 0.001) and preserved predictive accuracy (AUC 0.769). **Conclusions**: In PAD patients undergoing successful endovascular revascularization, long-term survival appeared to be related to systemic clinical factors and ischemic severity rather than anatomical lesion complexity. The GZ-PAD Mortality Score offers a simple and clinically applicable tool for mortality risk stratification. Further studies, including external validation in independent and multicenter cohorts, are needed to confirm the robustness and generalizability of the proposed risk score.

## 1. Introduction

Peripheral artery disease (PAD) represents an advanced clinical manifestation of systemic atherosclerosis and is associated with a markedly increased risk of mid and long-term mortality [1]. Despite advances in endovascular techniques and continuous improvements in limb-related outcomes, patients undergoing revascularization for PAD continue to experience mortality rates that remain significantly higher than those of the general population, suggesting that survival is driven by factors that extend beyond procedural success alone [1,2,3].

Several demographic and systemic clinical variables are identified to be related to mortality in patients with PAD. Moreover, the limb presentation with chronic limb-threatening ischemia (CLTI) and, in particular, the presence of tissue loss (Rutherford categories 5–6) is recognized as one of the highest-risk conditions for limb loss and mortality [2,4]. In contrast, the prognostic role of anatomical characteristics such as multisegment involvement, infrapopliteal disease and lesion complexity remains less clearly defined with respect to overall survival, despite their critical importance in guiding revascularization strategy and determining limb-related outcomes. However, most available studies have not comprehensively assessed the simultaneous contribution of all the abovementioned variables to mortality, nor have they translated this information into practical prognostic tools for routine clinical use.

Although several clinical predictors and selected risk models for mortality in patients with PAD have been previously described, most of them focus primarily on systemic comorbidities or cardiovascular risk, are not specifically designed for patients undergoing endovascular revascularization, and rarely integrate anatomical disease characteristics and limb clinical presentation within a single prognostic framework. Within this context, there is a clear need for simple and reliable predictive models capable of stratifying mortality risk in patients with PAD undergoing endovascular revascularization. Accordingly, the primary aim of the present study was to evaluate the impact of clinical-demographic variables, anatomical characteristics, and limb presentation on mid- to long-term mortality in a large real-world cohort. As a secondary objective, we sought to develop and internally validate a simple clinical risk score based on independent ischemic-systemic variables (the GZ-PAD Mortality Score) to facilitate individualized prognostic stratification in daily clinical practice.

## 2. Methods

### 2.1. Study Design and Population

**Figure 1:**
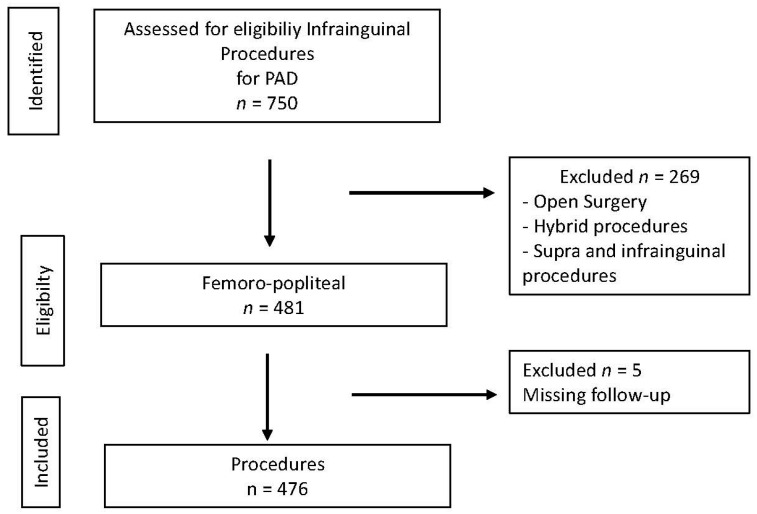
Flow diagram adapted for observational study. Number of procedures performed in patients suffering from peripheral arterial disease (PAD) in the study period.

We conducted a single-center, retrospective observational study over a 6-year period to identify patients suffering from symptomatic PAD who underwent endovascular revascularization as a first-line strategy. All patients were required to have complete clinical, anatomical and outcome data, which were extracted from institutional electronic medical records and procedural reports. The exclusion criteria included patients with missing follow-up data, primary open surgical or hybrid revascularization or repeated procedures. The unit of analysis was the individual patient; therefore, for patients undergoing multiple revascularizations, only the index procedure was considered for the purpose of the analysis. A flow diagram outlining patient selection and procedural stratification is presented in Figure 1.

Follow-up was performed in the outpatient setting and consisted of clinical and duplex ultrasound examination at 1, 3, and 6 months after the index procedure, and annually thereafter. Follow-up duration was calculated from the date of the index endovascular procedure to the occurrence of death or the last known clinical contact. Patients who did not experience the event of interest were censored at the date of their last available follow-up. Vital status was ascertained for all included patients through the institutional electronic record system. Five patients, residing outside the region, were excluded a priori, as detailed in the study flow diagram. As a result, the mortality data were available for all patients included in the final analysis, and no loss to follow-up occurred for the endpoint of all-cause mortality. Information on cause-specific mortality was not consistently available, as deaths occurring outside the hospital setting or in other institutions could not always be reliably adjudicated. For this reason, all-cause mortality was selected as the endpoint target of the study.

In addition, a prognostic model was developed and subsequently simplified into a practical point-based clinical score, the global ischemic-systemic variables (the GZ-PAD Score), to estimate individual mortality risk. The model and the clinical score were validated within the study cohort using survival stratification and ROC performance analysis.

### 2.2. Data Collection and Variable Definitions

The Strengthening the Reporting of Observational Studies in Epidemiology (STROBE) guidance was used during protocol design to ensure accurate reporting of study findings [5]. Baseline demographic, clinical, and procedural data were collected at the time of the index intervention. Collected variables included demographic characteristics and major clinical comorbidities. Limb clinical presentation before revascularization was recorded according to the Rutherford classification for PAD, with patients categorized as having intermittent claudication (Rutherford categories 2–3), rest pain (Rutherford category 4), or tissue loss (Rutherford categories 5–6).

Anatomical variables incorporated into the predictive model included femoropopliteal involvement, infrapopliteal disease (below-the-knee, BTK), multisegment femoropopliteal–tibial disease, and lesion complexity. Lesions were defined as complex when they exceeded 150 mm in length or in the presence of a chronic total occlusion (CTO), consistent with the TASC II classification system [6]. Preprocedural distal runoff was assessed, with runoff classified as poor in limbs with a single patent BTK vessel and good in limbs with two or three patent BTK vessels.

Anatomical characteristics were evaluated on intra-procedural angiography and recorded in the institutional database. The majority of procedures were performed by a single experienced operator, ensuring a high level of consistency in anatomical assessment and reducing interobserver variability. In addition, angiographic imaging was available for review during data collection and analysis, and any discrepancies were resolved by consensus among the authors.

### 2.3. Statistical Modeling and Score Development

Continuous variables were reported as mean ± standard deviation or median with interquartile range (IQR) as appropriate. Categorical variables were expressed as counts and percentages. Comparisons between survivors and non-survivors were performed using appropriate statistical tests according to data distribution and variable type. The association between baseline variables and all-cause mortality was evaluated using Cox proportional hazards regression.

Univariable Cox regression was performed for each baseline variable. Variables associated with all-cause mortality at univariable analysis (*p* < 0.10), together with clinically relevant covariates, were entered into a multivariable Cox proportional hazards model to identify independent predictors of mortality. Proportional hazards assumptions were assessed using Schoenfeld residuals. For each observation, the linear predictor (XBETA) was computed from the final multivariable Cox model coefficients and used as a continuous prognostic index. Patients were subsequently stratified into three prognostic categories based on the 33rd and 66th percentiles of the XBETA distribution. To enhance clinical applicability, regression coefficients from the final model were converted into a simplified point-based score (GZ-PAD Mortality Score), with points assigned proportionally to the magnitude of each predictor. Risk categories were defined according to the total point score. Discrimination between risk groups was assessed using Kaplan–Meier curves and the log-rank test. Hazard ratios (HRs) with 95% confidence intervals (CIs) were reported. A two-sided *p* value < 0.05 was considered statistically significant. To reduce the risk of overfitting, model complexity was limited by the inclusion of a restricted number of clinically relevant predictors. Internal validation was performed as a first step, and external validation is warranted.

Finally, to provide a comprehensive assessment of follow-up outcomes, Kaplan–Meier survival curves were constructed for clinically driven target lesion revascularization (cdTLR) and major adverse limb events (MALE). Clinically driven target lesion revascularization (cdTLR) was defined as any repeat endovascular or open intervention performed on the previously treated target vessel/lesion, in the presence of recurrent symptoms. Major Adverse Limb Events (MALE) were defined as the occurrence of major amputation of the index limb (above the ankle) or any repeat revascularization of the index limb, including both endovascular and surgical procedures, performed for recurrent ischemia due to restenosis, or failure of the initial revascularization. All MALE and cdTLR events were adjudicated with reference to the index limb corresponding to the baseline procedure.

Although MALE and cdTLR were systematically recorded during follow-up, they were not considered formal secondary endpoints of the study. Their analysis was performed to evaluate the long-term effectiveness and durability of the revascularization strategy, and to explore whether limb-related adverse events might have contributed to overall mortality. All analyses were performed using SPSS Statistics for Windows, Version V.28.0.1.1, (IBM Corp. Armonk, NY, USA).

## 3. Results

### 3.1. Baseline Population Characteristics

Over the study period, 476 patients undergoing endovascular treatment as first-line therapy for native femoropopliteal–tibial lesions were identified. The mean age of the cohort was 76.1 ± 10.4 years, and 40.8% of patients were female. More than half of the patients had a history of smoking, were affected by diabetes mellitus, and had chronic kidney disease.

With regard to anatomical disease distribution, nearly half of the patients required treatment limited to the femoropopliteal segment, while more than one-third presented with multisegment disease involving both the femoropopliteal and BTK arteries. Almost half of the cohort exhibited complex lesions. In addition, more than half of the treated limbs had poor distal run-off characterized by ≤1 patent BTK vessel. In regard to the limb presentation, approximately 66% of cases presented with tissue loss in the index limb. A detailed description of baseline characteristics is provided in Table 1. In all cases, endovascular revascularization was technically successful at the target vessel level, achieving restoration of direct inline flow from the common femoral artery to at least one distal runoff vessel. The median follow-up was 16 months [IQR 27], with a mean follow-up of 24.0 ± 18.7 months.

**Figure 2:**
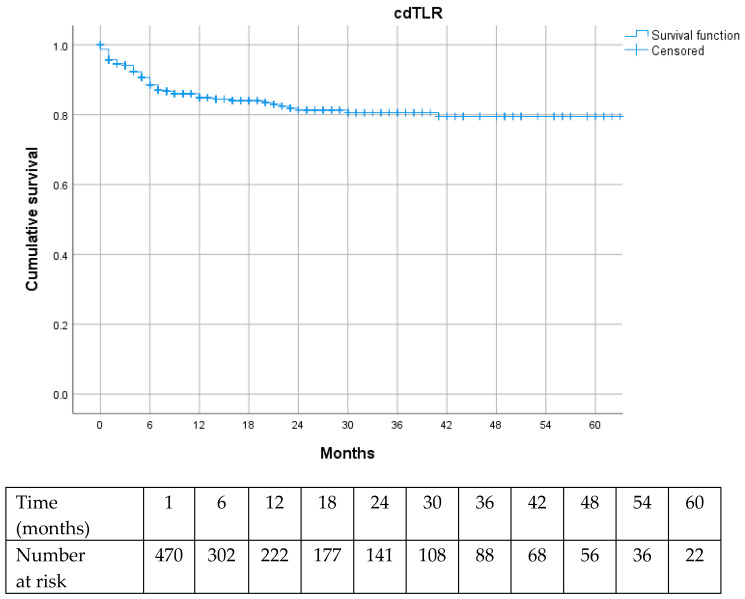
Kaplan–Meier curve for clinically driven target lesion revascularization (cdTLR).

**Figure 3:**
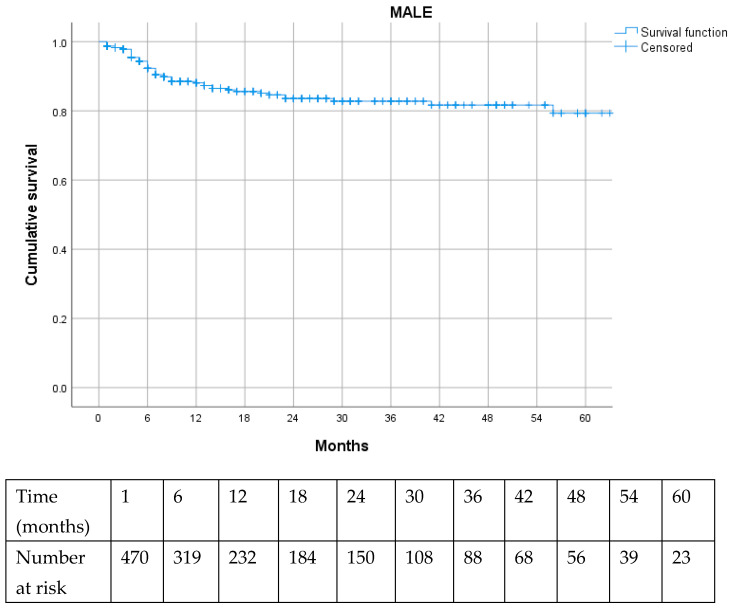
Kaplan–Meier curve for major adverse limb events (MALE) during follow-up.

Long-term outcomes in terms of reintervention rates, including cdTLR and MALE, were favorable, indicating durable effectiveness of the endovascular treatment (Figure 2 and Figure 3).

At univariable analysis, advanced age, CAD, CKD, dialysis dependence, and the severity of limb clinical presentation (rest pain and tissue loss) were strongly associated with mortality. In contrast, anatomical characteristics showed weak or non-significant associations with mortality, with the exception of multisegment disease involvement (Table 2).

Multivariable analysis. The final multivariable model identified age, CAD, CKD, dialysis dependence, and the presence of tissue loss (Rutherford categories 5–6) as independent predictors of all-cause mortality. After multivariable adjustment, several anatomical and clinical variables lost statistical significance, suggesting that long-term mortality was driven predominantly by systemic patient-related factors and by the clinical severity of limb ischemia, rather than by the anatomical complexity of arterial lesions (Table 3).

### 3.2. Development and Internal Validation of the Predictive Model

For each patient, the linear predictor (XBETA) was calculated from the coefficients of the final Cox regression model and used to derive the mortality score. Based on the 33rd and 66th percentiles of the XBETA distribution, patients were stratified into three prognostic categories: low risk (XBETA ≤ −0.43081), moderate risk (−0.43080 to 0.50835), and high risk (>0.50836).

**Figure 4:**
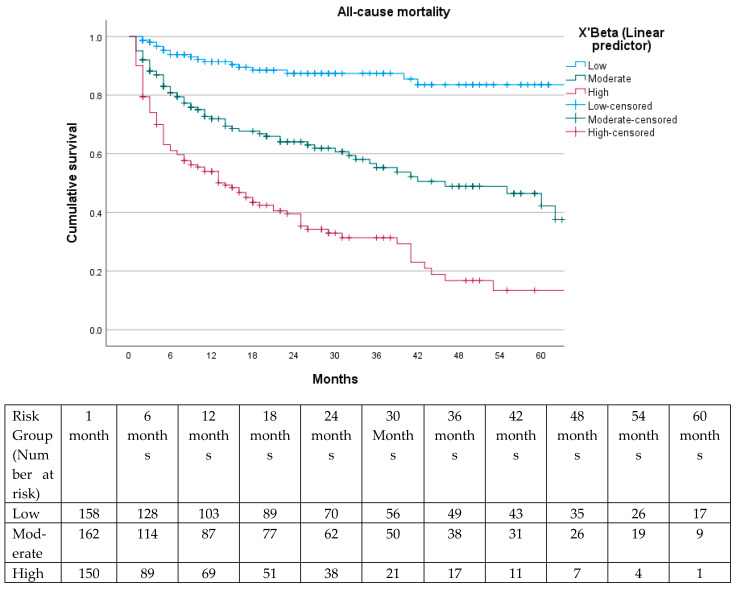
Kaplan–Meier survival curves for all-cause mortality according to the three prognostic categories defined by the Mortality Score (low, moderate, and high risk). A significant separation between risk groups was observed (log-rank *p* < 0.001).

**Figure 5:**
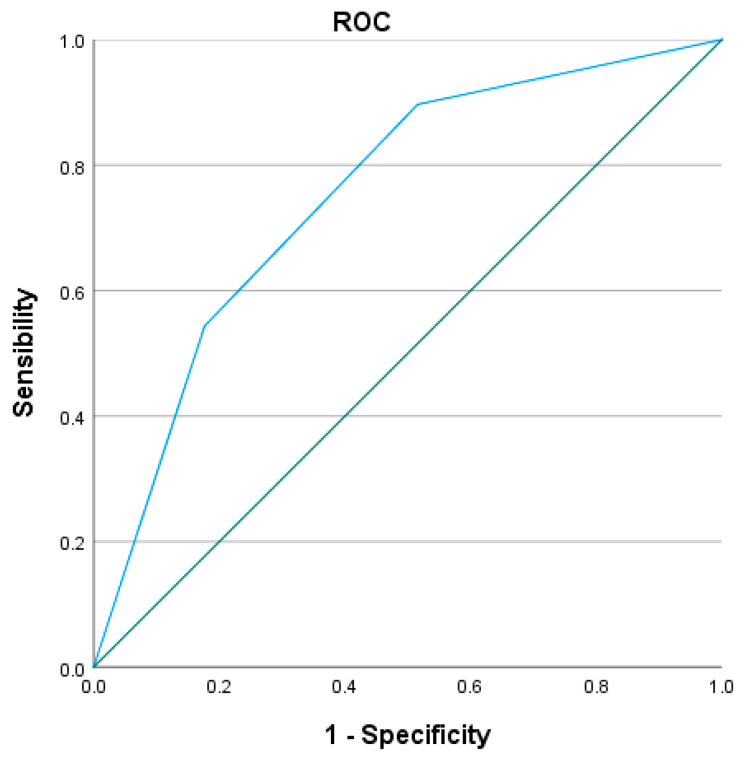
The receiver operating characteristic (ROC) curve of the XBETA-based model for all-cause mortality with AUC = 0.752 (95% CI 0.707–0.796; *p* < 0.001).

Subsequently, the prognostic relevance of the three XBETA-based risk categories was assessed using Kaplan–Meier survival curves, which demonstrated a highly significant separation between groups (log-rank χ^2^ = 102.441; *p* < 0.001) (Figure 4). ROC analysis demonstrated good discriminative ability of the model, with an area under the curve (AUC) of 0.752, indicating a robust capacity of the XBETA-based model to differentiate between survivors and non-survivors during follow-up (Figure 5).

### 3.3. Development of the Point-Based Clinical Score

The regression coefficients from the final multivariable model were converted into a simplified point-based clinical score, proportional to the prognostic weight of each independent predictor:

- Age 60–69 years = 1 point; 70–79 years = 2 points; ≥80 years = 3 points
- Coronary artery disease = 2 points
- Chronic kidney disease = 1 point
- Dialysis dependence = 3 points
- Tissue loss = 6 points

The total score allowed classification of patients into three prognostic categories:

Low risk: 0–6 points

Moderate risk: 7–10 points

High risk: ≥11 points

**Figure 6:**
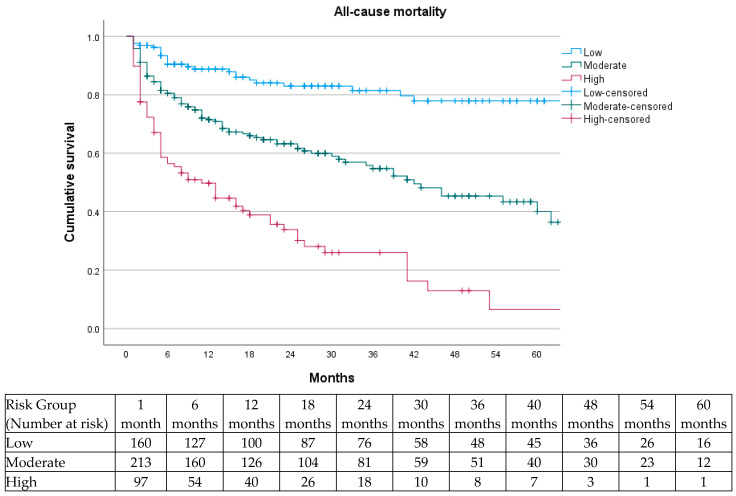
Kaplan–Meier survival curves for all-cause mortality according to the point-based GZ-PAD clinical score categories (low risk: 0–6 points; moderate risk: 7–10 points; high risk: ≥11 points). The score demonstrated excellent discrimination between risk strata (log-rank χ^2^ = 88.883; *p* < 0.001).

**Figure 7:**
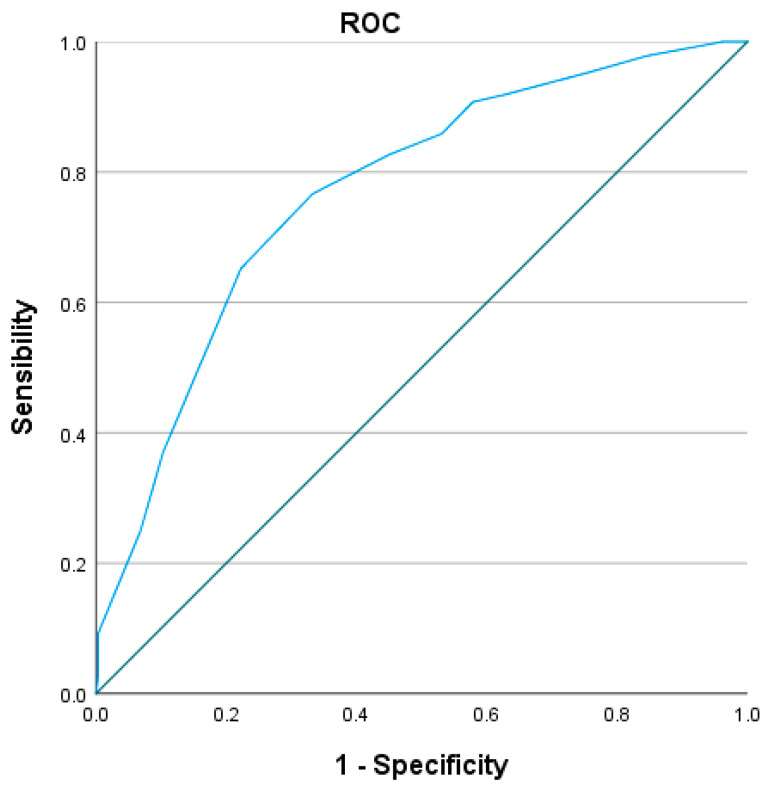
Receiver operating characteristic (ROC) curve of the simplified GZ-PAD clinical score for all-cause mortality with AUC = 0.769 (95% CI, 0.726–0.812; *p* < 0.001).

Internal validation using Kaplan–Meier analysis confirmed excellent discrimination between risk groups (log-rank χ^2^ = 88.883; *p* < 0.001), demonstrating the high clinical utility of the score. The prognostic model demonstrated good discriminative ability. ROC analysis yielded an AUC of 0.752 for the XBETA-based model and an AUC of 0.769 for the simplified GZ-PAD clinical score, indicating that the simplified score preserved the predictive accuracy of the full multivariable model (Figure 6 and Figure 7).

Moreover, the cumulative mortality was calculated from Kaplan–Meier survival at each follow-up interval for patients stratified into low-, moderate-, and high-risk groups based on the GZ-PAD clinical score (Table 4).

## 4. Discussion

In the present study, conducted in a real-world cohort of patients with PAD undergoing endovascular revascularization, medium- to long-term mortality was driven primarily by systemic patient-related factors and the clinical severity of limb ischemia, whereas many anatomical variables and lesion complexity parameters lost independent prognostic significance after multivariable adjustment. This finding is consistent with the current knowledge that considers PAD a manifestation of systemic atherosclerotic disease associated with a high burden of intrinsic cardiovascular mortality [1,2,4]. Advanced age, coronary artery disease, advanced renal dysfunction, and particularly dialysis dependence emerged as dominant determinants of survival. Multiple studies have demonstrated that patients with PAD and end-stage renal disease experience extremely high mortality rates, often exceeding the prognostic impact of anatomical lesion characteristics [7,8,9]. Similarly, in addition to advanced age, coronary artery disease is a well-recognized marker of systemic cardiovascular instability that profoundly influences overall survival [10,11].

Moreover, tissue loss (Rutherford categories 5–6) was confirmed as the most powerful predictor of mortality in our multivariable model. This finding is consistent with the recommendations of the European Society for Vascular Surgery (ESVS), which identifies CLTI with tissue loss as a condition of extremely high systemic risk rather than merely a local limb disorder [1,2]. Tissue loss reflects an advanced stage of systemic atherosclerotic disease, commonly associated with chronic inflammation, recurrent infections, malnutrition, and multiorgan dysfunction [12,13,14].

Furthermore, anatomical complexity was included in the analysis because it may reflect the overall burden and stage of atherosclerotic disease and may be associated with greater procedural complexity and potentially influence mortality indirectly. A central interpretative finding of our study is that, following technically successful endovascular revascularization, many anatomical variables, including multisegment femoropopliteal–tibial involvement and lesion complexity, lose their independent prognostic impact on mortality. This observation suggests that the uniformly high technical success of endovascular revascularization in this cohort may have attenuated the prognostic impact of anatomical complexity on long-term survival, making it predominantly dependent on the patient’s systemic risk profile. It should be noted that anatomically advanced disease was well represented in the present cohort, suggesting the robustness of the findings despite specific formal interaction testing between systemic comorbidities and anatomical characteristics not being performed.

Numerous studies have established that anatomical complexity is a major determinant of revascularization strategy selection and technical success, and that it strongly influences limb-related outcomes, including primary patency, cdTLR, and MALE [15,16,17,18]. In particular, limb loss is recognized to be associated with a high mortality rate during the follow-up and strongly associated with tissue loss presentation [1,2]. In our cohort, long-term limb outcomes were remarkably favorable, with freedom from both cdTLR and MALE approaching 80% at 60 months of follow-up. Despite their low incidence, the potential impact of these events on long-term mortality warrants further evaluation; however, longer follow-up is required to obtain robust data, and this will be the focus of future investigations in our cohort.

On this basis, the simple clinical prognostic score developed relies exclusively on systemic patient-related factors and the severity of limb presentation. The clear separation of Kaplan–Meier survival curves across the three risk categories (*p* < 0.001), together with the ROC analysis, demonstrates that the model provides clinically meaningful prognostic stratification. Such a model aligns with current recommendations that emphasize the integration of systemic risk and CLTI severity into therapeutic decision-making [1,2].

Taken together, our findings reinforce the concept that the management of advanced PAD should evolve from a purely “lesion-centered” approach toward a “patient-centered” strategy, in which limb revascularization represents only one component of a comprehensive risk management [19,20,21]. The main strengths of this study include the large cohort size, the availability of detailed clinical and anatomical variables, the use of a time-to-event analytical approach with Cox regression modeling, and the derivation of a simple and clinically applicable risk score with internal validation based on Kaplan–Meier stratification and log-rank testing.

### Limitations

This study has inherent limitations related to its retrospective and single-center design, with potential selection bias and residual confounding. In addition, the impact of technical failure and the incidence of cdTLR and MALE on mortality were not formally evaluated. However, in our cohort, these events occurred at relatively low rates and were therefore considered surrogate indicators of effective and durable revascularization. Another limitation is that the risk score was developed exclusively in patients treated with endovascular techniques and thus does not account for the potential impact of open surgical revascularization strategies. Finally, although the score demonstrated good discrimination in the present cohort, external validation in independent cohorts is required to assess its generalizability, calibration, and predictive performance across different clinical settings. The lack of systematic information on cause-specific mortality limits the ability to explore cause-specific mechanisms of death but does not affect the validity of all-cause mortality as the primary study endpoint.

## 5. Conclusions

In a cohort of patients with PAD undergoing successful endovascular revascularization, long-term survival appeared to be more strongly associated with the patient’s systemic risk profile than with lesion complexity. Further studies, including external validation in independent and multicenter cohorts, are needed to confirm the robustness and generalizability of the proposed risk score.

## Figures and Tables

**Table 1:** Baseline clinical-demographic and anatomical characteristics of the study population. For each category, the total number of patients is reported, and in parentheses, the number and percentage of deaths observed within that category. *p* values were calculated using contingency tables (χ^2^ test or Fisher’s exact test, as appropriate) and are intended for descriptive purposes only. * runoff assessed prior to the procedure.

	No (%) Tot = 476	Mortality N (%)	*p* Value
Age (median ± SD)	76.13 ± 10.4		
Female	194 (40.8%)	81 (41.8%)	0.250
Smoking	282 (59.2%)	91 (32.3%)	<0.001
Diabetes	282 (59.2%)	114 (40.4%)	0.339
CAD	159 (33.4%)	80 (50.3%)	<0.001
Hypertension	423 (88.9%)	169 (40.0%)	0.101
FA	141 (29.6%)	77 (54.6%)	<0.001
CKD	272 (57.1%)	145 (53.3%)	<0.001
Dialysis	47 (9.9%)	33 (70.2%)	<0.001
COPD	67 (14.1%)	27 (40.3%)	0.766
Dyslipidemia	314 (66%)	128 (40.8%)	0.188
CVD	65 (13.7%)	32 (49.2%)	0.060
Neoplasia	101 (21.2%)	40 (39.6%)	0.825
Obesity	91 (19.1%)	35 (38.5%)	0.966
Fem-pop	241 (50.6%)	72 (29.9%)	<0.001
BTK	78 (16.4%)	30 (38.5%)	0.969
Fem-pop-BTK	163 (34.2%)	84 (51.5%)	<0.001
Complex lesion (>150. CTO)	223 (46.8%)	80 (35.9%)	0.192
Claudication (Rutherford 2–3)	66 (13.9%)	6 (9.1%)	<0.001
Rest pain (Rutherford 4)	93 (19.5%)	23 (24.7%)	0.002
Tissue loss (Rutherford 5–6)	317 (66.6%)	155 (48.9%)	<0.001
Quality runoff *	256 (53.7%)	81 (31.6%)	<0.001

**Table 2:** Univariable Cox regression survival analysis of clinical-demographic and anatomical variables associated with mortality. Hazard ratios (HRs); 95% confidence intervals (95% CIs); and *p* values are reported. An HR > 1 indicates an increased risk of mortality, whereas an HR < 1 indicates a reduced risk compared with the reference category. Each variable was analyzed individually as a predictor of all-cause mortality during follow-up. * runoff assessed prior to the procedure.

	HR	95% CI	*p* Value
Age (median ± SD)	1.041	1.025–1.057	<0.001
Female	1.156	0.864–1.546	0.330
Smoking	0.660	0.494–0.882	0.005
Diabetes	1.199	0.890–1.615	0.232
CAD	1.979	1.477–2.651	<0.001
Hypertension	1.539	0.907–2.609	0.110
FA	2.019	1.505–2.707	<0.001
CKD	2.954	2.073–4.208	<0.001
Dialysis	2.449	1.677–3.576	<0.001
COPD	1.105	0.734–1.663	0.633
Dyslipidemia	1.317	0.962–1.804	0.086
CVD	1.478	1.010–2.165	0.045
Neoplasia	1.313	0.923–1.868	0.130
Obesity	0.829	0.573–1.200	0.320
Fem-pop	0.664	0.494–0.894	0.007
BTK	0.932	0.630–1.379	0.725
Fem-pop-BTK	1.615	1.208–2.160	0.001
Complex lesion (>150. CTO)	0.967	0.704–1.327	0.834
Claudication	0.184	0.082–0.416	<0.001
Rest pain (Rutherford 4)	0.523	0.337–0.809	0.004
Tissue loss (Rutherford 5–6)	3.137	2.108–4.668	<0.001
Quality runoff *	0.654	0.489–0.875	0.004

**Table 3:** Multivariable Cox regression analysis of factors associated with all-cause mortality. Hazard ratios (HRs), 95% confidence intervals (95% CIs), and *p* values are reported. An HR > 1 indicates an increased risk of mortality, whereas an HR < 1 indicates a reduced risk compared with the reference category. The model was adjusted for all variables included in the table.

Variables	HR [Exp(B)]	95% CI	*p*-Value
Age	1.041	1.021–1.061	<0.001
CAD	1.560	1.132–2.149	0.007
CKD	1.523	1.023–2.268	0.038
Dialysis	2.500	1.613–3.874	<0.001
Tissue loss (Rutherford 5–6)	5.333	2.308–12.325	<0.001

**Table 4:** Cumulative mortality rates over time according to GZ-PAD score categories.

	Mortality Rate (%)					
Risk Group	6 months	12 months	24 months	36 months	48 months	60 months
Low	9	12	13	14	23	23
Moderate	18	27	33	42	55	58
High	52	50	66	74	87	94

## Data Availability

The original contributions presented in this study are included in the article/Supplementary Material. Further inquiries can be directed to the corresponding author.

## Supplementary Materials

The following supporting information can be downloaded at: https://www.mdpi.com/article/10.3390/jcm15041364/s1, STROBE Checklist—GZ-PAD Mortality Study.

## Abbreviations

The following abbreviations are used in this manuscript:

PAD	peripheral arterial occlusive disease
PAD	peripheral arterial disease
CAD	coronary artery disease
FA	atrial fibrillation
CKD	chronic kidney disease;
CVD	cerebrovascular disease
COPD	chronic obstructive pulmonary disease
Fem-pop	femoro-popliteal
BTK	below-the-knee
CTO	chronic total occlusion
cdTLR	clinically driven target lesion revascularization
MALE	Major adverse limb events
HR	hazard ratio
CI	confidence interval
ROC	Receiver operating characteristic
