# Supplementary material for: Predictors of Mortality in Peripheral Arterial Disease After Endovascular Lower Limb Revascularization and Development of a Risk Score Based Solely on Clinical Presentation
Source: J Clin Med. 2026 Feb 9;15(4):1364. doi: 10.3390/jcm15041364 (PMC12941924; doi:10.3390/jcm15041364)
Supplement: Supplementary file 1 [file jcm-15-01364-s001.zip › jcm-4110214-supplementary.pdf]

STROBE Checklist – GZ-PAD Mortality Study

No.	Section	Recommendation	Manuscript location	Status	Notes
1a	Title	Indicate study design in title/abstract	Abstract – Methods	Yes	Retrospective cohort stated
2	Background	Scientific background and rationale	Introduction	Yes	
3	Objectives	Specific objectives	Introduction	Yes	
4	Study design	Key elements early	Methods—Design	Yes	Single-centre retrospective cohort
5	Setting	Setting, dates, follow-up	Methods—Setting/Follow-up	Yes	
6a	Participants	Eligibility criteria	Methods—Participants	Yes	
7	Variables	Define outcomes/exposures	Methods—Definitions	Yes	
8	Data sources	Sources/measurement	Methods	Yes	
9	Bias	Address potential bias	Methods; Discussion	Yes	
10	Study size	How arrived at	Results—Flow	Yes	
11	Quantitative	Handling of variables	Methods	Yes	
12a	Statistics	Confounding control	Methods—Statistics	Yes	
13	Participants	Numbers at each stage	Results: Figure 1	Yes	

14	Descriptive	Participant characteristics	Results: Table 1	Yes
15	Outcome	Events over time	Results; Figures	Yes
16	Main results	Adjusted/unadjusted	Results: Table 3	Yes
18	Key results	Summarised vs objectives	Discussion	Yes
19	Limitations	Study limitations	Discussion	Yes
20	Interpretation	Overall interpretation	Discussion	Yes
21	Generalisability	External validity	Discussion	Yes
22	Funding	Source and role	Funding/COI	Yes
